# The large numbers of minicolumns in the primary visual cortex of humans, chimpanzees and gorillas are related to high visual acuity

**DOI:** 10.3389/fnana.2022.1034264

**Published:** 2022-11-09

**Authors:** Mark N. Wallace, Oliver Zobay, Eden Hardman, Zoe Thompson, Phillipa Dobbs, Lisa Chakrabarti, Alan R. Palmer

**Affiliations:** ^1^Medical Research Council (MRC) Institute of Hearing Research, University Park, Nottingham, United Kingdom; ^2^Hearing Sciences, Mental Health and Clinical Neurosciences, School of Medicine, University of Nottingham, Nottingham, United Kingdom; ^3^School of Medicine, University of Nottingham, Hearing Sciences—Scottish Section, Glasgow Royal Infirmary, Glasgow, United Kingdom; ^4^Veterinary Department, Twycross Zoo, East Midland Zoological Society, Atherstone, United Kingdom; ^5^School of Veterinary Medicine and Science, University of Nottingham, Sutton Bonington Campus, Nottingham, United Kingdom

**Keywords:** ferret, rodent, guinea pig, dendritic bundles, pyramidal cells, axonal bundles, hedgehog (*Erinaceus europaeus*)

## Abstract

Minicolumns are thought to be a fundamental neural unit in the neocortex and their replication may have formed the basis of the rapid cortical expansion that occurred during primate evolution. We sought evidence of minicolumns in the primary visual cortex (V-1) of three great apes, three rodents and representatives from three other mammalian orders: Eulipotyphla (European hedgehog), Artiodactyla (domestic pig) and Carnivora (ferret). Minicolumns, identified by the presence of a long bundle of radial, myelinated fibers stretching from layer III to the white matter of silver-stained sections, were found in the human, chimpanzee, gorilla and guinea pig V-1. Shorter bundles confined to one or two layers were found in the other species but represent modules rather than minicolumns. The inter-bundle distance, and hence density of minicolumns, varied systematically both within a local area that might represent a hypercolumn but also across the whole visual field. The distance between all bundles had a similar range for human, chimpanzee, gorilla, ferret and guinea pig: most bundles were 20–45 μm apart. By contrast, the space between bundles was greater for the hedgehog and pig (20–140 μm). The mean density of minicolumns was greater in tangential sections of the gorilla and chimpanzee (1,243–1,287 bundles/mm^2^) than in human (314–422 bundles/mm^2^) or guinea pig (643 bundles/mm^2^). The minicolumnar bundles did not form a hexagonal lattice but were arranged in thin curving and branched bands separated by thicker bands of neuropil/somata. Estimates of the total number of modules/minicolumns within V-1 were strongly correlated with visual acuity.

## Introduction

Neocortical minicolumns are thought to be fundamental morphological units (Mountcastle, 1978; Buxhoeveden and Casanova, 2002b) and are formed by a narrow chain of interconnected neurons extending radially across layers II–VI. Their structure may have evolved over the last 70 million years to facilitate specialized or efficient use of neural computation (Buxhoeveden and Casanova, 2002a; Raghanti et al., 2010). They may represent a specialized form of the repeating modules or microcolumns that have similar widths, but are usually restricted to one or two cortical layers (Buldyrev et al., 2000; Jones, 2000). The evolutionary increase in computational power that comes from bigger brains (Kaas, 2005) is thought to come from an iterative replication of these minicolumns (Rakic, 1995, 2008; Chenn and Walsh, 2002) during the recent cortical expansion that has been particularly prominent in primates over the last 10 million years (Buxhoeveden and Casanova, 2002a; Blazek et al., 2011). The diameter of the minicolumns and the number and type of neurons within them varies across species (Buxhoeveden and Casanova, 2002a; Yanez et al., 2005; Herculano-Houzel et al., 2008). However, the presence of minicolumns has still not been established in all cortical areas or in all species.

**Figure 1:**
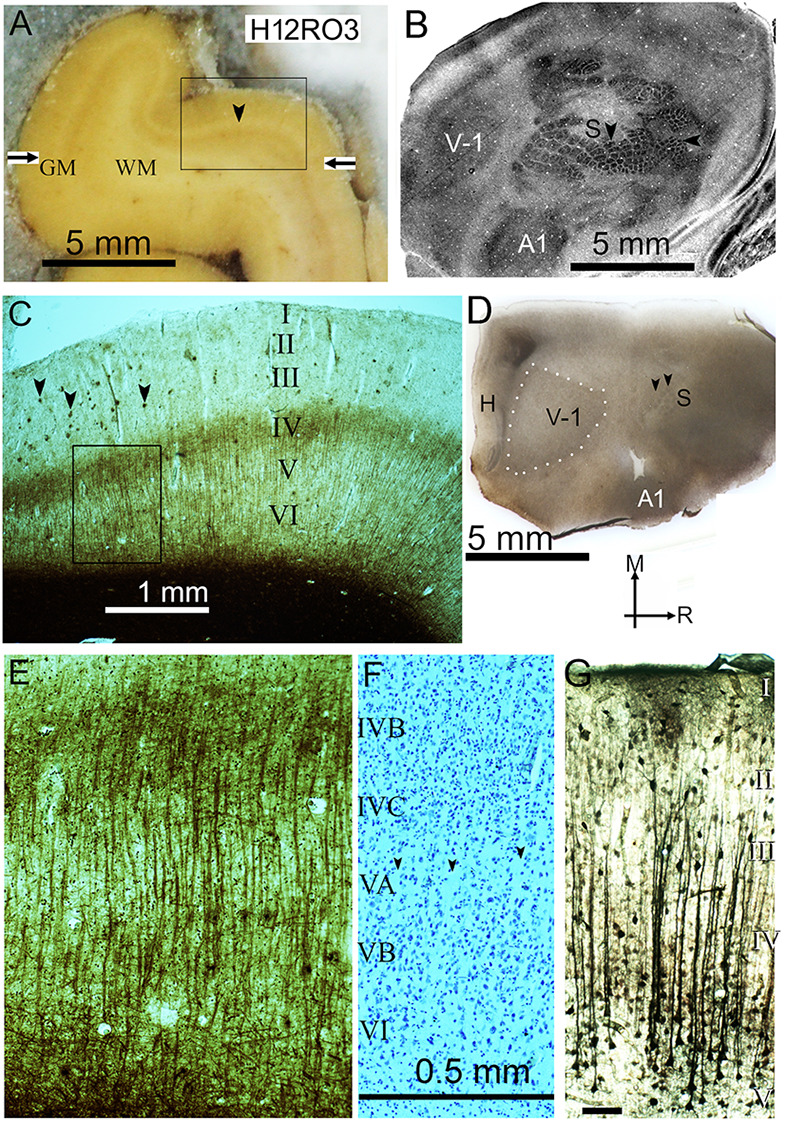
**(A)** Frozen block of visual cortex from the third block of the right occipital lobe in the second human brain (H12RO3), on the microtome stage during sectioning. The gray matter (GM) contains more fixed blood than the paler white matter (WM) and the pale line that forms the stria of Gennari (arrowhead) defines the primary visual cortex. The borders of the primary visual area are marked by the horizontal arrows. The rectangular box indicates the position of the section shown in panel **(C)**. **(B)** Surface parallel section through the flattened rat neocortex stained for cytochrome oxidase activity. The section mainly passes through layer IV and sharp changes in the enzyme activity help to define the primary visual (V-1), primary somatosensory (S) and primary auditory (A1) areas. Within the somatosensory area there are distinct barrel-like structures that correspond to the input from individual whiskers (vertical arrowhead) or guard hairs (horizontal arrowhead) as described previously (Wallace, 1987). **(C)** Myelin-stained section from the position indicated in panel **(A)**, showing the stria of Gennari in the middle of layer IV. There are small pathological clumps of stained material in the outer layers (marked by arrowheads) that correspond to beta amyloid plaques but they are not present in significant numbers in the deep layers. The rectangular box shows the position of the image shown at higher power in panel **(E)**. **(D)** Flattened tangential section of rat cortex stained for myelin. This shows the location of the three main sensory areas as well as the hippocampus (H). The section passes through layer IV of the primary visual (V-1) and posterior somatosensory (S) areas and obliquely through the primary auditory area (A1) at the lateral edge of the block. The primary visual area is outlined by white dots while the two black arrow heads indicate the position of whisker barrels. The arrows indicate the medial (M) and rostral (R) directions. **(E)** Higher power view of the part of the section indicated in panel **(C)** that shows the parallel bundles of myelinated fibers that are characteristic of the deep layers of the primary visual cortex. **(F)** Corresponding area of a nearby section stained for Nissl substance. There is some evidence of pale lines of neuropil (see arrowheads) that lack any somata and define thin minicolumns of cells in layer V but they are not as clear or well-defined as the myelin bundles. The laminae are denoted by the Roman numerals and also apply to panel **(E)**. **(G)** Coronal section of the guinea pig auditory cortex showing bundles of apical dendrites from layer V pyramidal cells that show high thallium uptake. The same scale bar applies for **(E,F)** and the bar in **(G)** represents 0.1 mm.

The first physiological evidence for columns was found in the cat somatosensory cortex (Mountcastle, 1957) and subsequent physiological studies showed a honeycomb-like mosaic with vertical columns stretching through the cortical layers and having a diameter of 350–400 μm (Favorov et al., 1987; Favorov and Diamond, 1990). Before this Lorente de No had identified evidence of repeating modular units and identified the importance of vertical connections in the mouse somatosensory cortex (Lorente de No, 1922). These were subsequently shown to be part of the columns based on the mystacial whisker barrels that can be conveniently studied by simple histochemical methods (Woolsey and Van der Loos, 1970; Wong-Riley and Welt, 1980) Figure 1B (Wallace, 1987). The barrels are restricted to layer IV, while minicolumns, by physiological definition (Mountcastle, 1997), stretch across all six layers. Each barrel appears to contain many physiologically defined modules containing neurons preferring the same whisker deflection angle (Bruno et al., 2003) but it has been difficult to identify corresponding entities using morphological methods. There are bundles of apical dendrites present in the rodent somatosensory cortex but in the mouse they only contain a mean value of 5.4 dendrites per bundle (Smit-Rigter et al., 2011) and although there are myelinated axons, which are denser in the barrel hollows than the walls of the rat (Figure 1D), the fibers are not organized into clear bundles that would correspond to minicolumns (Land and Erickson, 2005). In mice at least they seem to represent thalamocortical fibers rather than efferent fibers (Barrera et al., 2013). More importantly, whisker barrels have not been found in primates or carnivores and they become less prominent or are not present in large rodents such as the capybara (Woolsey et al., 1975). This makes it difficult to compare minicolumns across different mammalian orders in the primary somatosensory area.

Evidence of minicolumns in the auditory region is particularly interesting because of their potential involvement in language and the lateralization between their sizes in the human planum temporale which is not found in the chimpanzee or rhesus monkey (Buxhoeveden and Casanova, 2000). These minicolumns are formed from radial strings of neurons associated with bundles of myelinated fibers and apical dendrites (Buxhoeveden and Casanova, 2002b) but the evidence for minicolumns in the auditory cortex of non-primates is relatively weak (Wallace and He, 2011). One way in which it is possible to link bundles of apical dendrites to functional columns is with a histological technique that indicates the level of functional activity by measuring the active uptake of potassium ions using thallium ions to mimic them. Using thallium autometallography, bundles of apical dendrites were shown at the center of a column of high uptake produced by tonal stimulation in the gerbil auditory cortex (Goldschmidt et al., 2004). Using an improved method for thallium autometallography (Goldschmidt et al., 2010), we found the presence of long bundles of apical dendrites arising mainly from layer V pyramids and extending to the base of layer II in the auditory cortex of urethane anesthetized guinea pigs (Figure 1G). These bundles were about 60 μm from center-to-center but contained variable numbers of dendrites and were only a single subset of the bundles that would be expected in the cortex of an alert animal. Moreover, functional techniques of this type are only really suitable for laboratory animals and it is difficult to make exact homologies between the auditory areas coming from species of different mammalian orders (Kaas, 2011). The primary visual area (V-1) was a much more promising area for comparative studies.

In his ground-breaking study of the architectonic parcellation of cortex in a wide range of mammalian species, from the hedgehog to human, Brodmann identified V-1 as the area that can be most reliably identified across a wide range of species (Brodmann, 1909) and this is still accepted today (Fischl and Sereno, 2018). A primary visual cortex has been demonstrated in every mammalian species where it has been studied (Rosa and Krubitzer, 1999). Physiological evidence for minicolumns was first obtained in the V-1 of the cat and monkey (Hubel and Wiesel, 1977). However, for protected great apes and generally in the human, invasive electrophysiological methods are not possible. Instead, anatomical methods suitable for archival brain tissue are used instead. The most basic anatomical method for studying minicolumns is by identifying lines of Nissl-stained cells in sections orthogonal to the surface Figure 1F (Buxhoeveden and Casanova, 2002b; Casanova et al., 2008). However, lines of neurons cannot be identified in tangential sections and a more reliable method for measuring the density of minicolumns is to study the bundles of myelinated axons (Meynert's bundles) found in most regions of the human (Nieuwenhuys, 2013) and the chimpanzee neocortex (Strasburger, 1937). Bundles of myelinated axons are not present in all neocortical areas, but are particularly clear in V-1 (Figure 1E). The primary advantage of using the myelinated bundles is that they can be equally well-identified in both orthogonal and tangential sections. Secondly, the fiber bundles never bifurcate (Casanova et al., 2008) unlike the bundles of apical dendrites where dendritic branches can split off to join other bundles (Hosoya, 2019). The most detailed description of the structural basis of minicolumns so far uses data from the macaque monkey V-1 (Peters and Sethares, 1996). Each radial minicolumn contained pyramidal neurons which formed a single bundle of apical dendrites and had a single bundle of descending myelinated axons. These output axons from the pyramidal cells contained 34 ± 13 axons and entered the white matter. In the primate brain there are also radial bundles of intrinsic axons formed by a regular array of inhibitory interneurons called double bouquet cells. These axons cross over a number of layers and appear to be interdigitated between the bundles of apical dendrites (del Rio and DeFelipe, 1997). They are mainly found in the primate and carnivore brain and have not been identified in other mammalian orders (Yanez et al., 2005). However, rodent V-1 does contain apical dendritic bundles with a mean of about seven dendrites (range 2–70) and the distance between them is about 50 μm (Innocenti and Vercelli, 2010). Their unifying feature may be the presence of a common target as shown by retrograde labeling (Vercelli et al., 2004).

We now address the important problem of establishing, whether or not, similar minicolumns exist in the same area across a wide range of mammalian orders, for the first time in a single study. We used a simple but reliable method for clearly identifying minicolumns following a standardized protocol for staining myelinated fibers. We aimed to comprehensively study the geometry of the radial bundles of myelinated fibers in V-1 to better understand the potential role of the minicolumns that they represent. By comparing the bundles in representative species from five mammalian orders we wanted to confirm whether or not evidence of minicolumns was present in all species. We also sought to relate the bundle characteristics to other aspects of the visual system such as the total surface area of V-1, the total number of neurons in V-1 and visual acuity. It has previously been suggested that visual acuity is linked to computational power in terms of the numbers of neurons in V-1 (Srinivasan et al., 2015) but we wanted to test the hypothesis that neural computation was more closely linked to the numbers of basic neural circuits (minicolumns or microcolumns) in an area rather than the total numbers of neurons.

## Materials and methods

### Tissue samples and preparation

Samples of two male human brains were obtained from the Nottingham Health Sciences Biobank. There were no neurological problems, but pathological examination showed a low incidence of tau tangles in the temporal lobe of the first brain and a low number of beta amyloid plaques in the cortex of the second brain, including V-1 (Figure 1C). Silver staining methods such as those developed by Gallyas for studying myelin can also show pathological changes such as beta amyloid plaques (Uchihara, 2007). The first brain was from a 58-year-old man (H11D25694) who died of sepsis following immunosuppression to allow treatment for multiple myeloma. The second was from an 81-year-old man (H12D12468) who died of bladder cancer and ischaemic heart disease. The brains were removed intact during post-mortem examination and fixed by suspension in 10% formalin. The human brains were archival material which had been in formalin for between 1 and 8 years.

All the animal brains were removed from the cranium and fixed by immersion in 10% formalin to standardize the amount of shrinkage. The V-1 area (area 17) and adjacent areas were obtained from eight mammalian species so that a comparison could be made with area 17 in the human. Cortical samples were taken at postmortem from two great apes (Hominidae family): *Pan troglodytes* (two female and one male) and *Gorilla gorilla* (two males) which had been part of the large primate collection at Twycross zoo (https://twycrosszoo.org/) and had died of age-related natural causes. The ape brains were archival material which had been in formalin for between 1 and 8 years. Three common laboratory rodents were used: *Cavia porcellus* (two male and two female; tricolor, bred in house)*, Rattus rattus* (two female and one male; Wistar, albino) and *Mus musculus* (CBA strain, five male and three female; Charles River) and three species were from separate mammalian orders to give a wide phylogenetic spread. These were a single female European hedgehog (*Erinaceus europaeus*) from the Eulipotyphla, a domestic pig (*Sus scrofa domesticus*) from the Artiodactyla and four ferrets (*Mustela putorius*, two male and two female, sable, bred in house) from the Carnivora. The human and ape brains were obtained at post-mortem, at an unrecorded period after death (usually <24 h) and this was also true of the hedgehog and pig. The brains of the ferrets and rodents were all removed immediately after death and fixed by immersion in 10% formalin without any phosphate buffer that would usually be used. Specimens were not age matched but all had reached maturity. The hedgehog (female weighing 767 g) had been killed accidentally by being run over and was found at the side of the road near Penrith (England). There was no sign of rigor mortis and it was estimated that <24 h had elapsed between death and immersion in fixative. The pig brain came from a local abattoir.

The V-1 area was identified by its distinctive myelin staining. In the three primate brains the stria of Gennari was used in preparing blocks for sampling in the orthogonal and tangential planes relative to the surface (Figure 1A). For the other brains, atlases were used in removing blocks for sampling: pig brain (Saikali et al., 2010), rat brain (Paxinos and Watson, 1986), The Allen Brain Atlas for the Mouse (https://mouse.brain-map.org/static/atlas) or reference was made to physiological studies of V-1 in that species: hedgehog (Kaas et al., 1970), guinea pig (Choudhury, 1978) and ferret (Law et al., 1988). The visual cortex from all the brains was cut into slabs or blocks that were no more than 2 cm thick and then submerged again in 10% formalin for a minimum of 48 h. These blocks were subsequently cryoprotected through immersion in 30% sucrose for a minimum of 48 h and until they were no longer buoyant. None of the brains was fixed by perfusion so that the degree of shrinkage was kept constant. Sections were cut at 40 μm and post fixed in 10% formalin before being mounted from distilled water on Superfrost plus slides (https://www.fishersci.co.uk/shop/products/superfrost-plus-adhesion-slides-9/10149870) and air dried. Sections were then stained for myelinated fibers with the Gallyas silver stain (Gallyas, 1979) or using a standard Cresyl Violet stain for Nissl substance.

### Histological staining

Most sections were stained with the Gallyas method for myelin and were dehydrated in a graded alcohol series before immersion in a solution of 150 ml pyridine and 75 ml glacial acetic acid for 30 min. The sections were then washed quickly in three changes of distilled water before pre-incubation (1 h) in a solution of 200 mg ammonium nitrate, 200 ml distilled water, 200 mg silver nitrate and 1 ml of 4% sodium hydroxide (stirred vigorously and in that order). After three rinses in 0.5% acetic acid (3 min each) they were placed in the physical developer made up of three stock solutions: (A) 25 g of anhydrous sodium carbonate in 500 ml of distilled water; (B) 400 mg ammonium nitrate, 400 mg silver nitrate, 2 g silicotungstic acid dissolved in 200 ml of distilled water; (C) 1 g ammonium nitrate, 1 g silver nitrate, 5 g silicotungstic acid dissolved in 36.5 ml of 4% paraformaldehyde (without buffer) in 463.5 ml of distilled water. The developer was made up by combining 100 ml of A, 20 ml of B and 80 ml of C while stirring vigorously. Sections were then rinsed once in 0.5% acetic acid followed by three quick rinses in distilled water. The background was cleared by placing them in 0.2% potassium ferricyanide and fixed in 0.5% sodium thiosulphate (1 min.) before rinsing in distilled water. Sections were air dried, dehydrated in alcohols and mounted in Entellan mounting medium (https://www.sigmaaldrich.com/GB/en/product/mm/107960). Additional sections were stained for Nissl substance using a 0.5% solution of Cresyl Violet in acetate buffer (pH 3.9).

An additional two guinea pig brains were prepared for thallium autometallography to determine if it would be a suitable method for identifying bundles of apical dendrites. Coronal sections of the guinea pig neocortex were sectioned after the brain had been loaded with chelated thallium ions to indicate the levels of potassium uptake and associated neuronal activity (Goldschmidt et al., 2010). To prepare the brain for staining, two animals were terminally anesthetized with urethane and the external jugular vein was cannulated. A freshly prepared solution of thallium chelated with diethyldithiocarbamate, consisting of 3 ml of a 0.2% suspension, was injected into the vein over a period of 3 min and then the canula flushed through with physiological saline. The animals were given a lethal dose of pentobarbitone (i.v.) 2 min later to limit the amount of thallium uptake and the brains prepared for autometallography as described previously (Goldschmidt et al., 2010; Coomber et al., 2011). Frozen sections (40 μm) were cut in the coronal plane, on the sledge microtome.

### Histological analysis

Images were obtained using a Zeiss AXIO II microcam and analyzed using the commercial neuron tracing software Neurolucida (https://www.mbfbioscience.com/neurolucida). The distance between the center point of myelin bundles was measured at the upper edge of cortical layer V in transverse sections using a function for measuring the distance between two points. No automated algorithm was used and every data point was measured manually using a definition of a bundle based on the judgement of the researcher. In tangential sections, areas were chosen for analysis where the bundles were sectioned in a plane orthogonal to the long axis of the bundles and bundles were judged to be in layer V or the base of layer IVC when the section was clearly below the stria of Gennari. The center of each bundle was plotted as a point using the Neurolucida programme and then exported as a set of 3 dimensional coordinates into an Excel spreadsheet.

The uniformity (or otherwise) of the local density of bundles across the whole extent of tangential sections was assessed by estimating the spatially varying local point density. This was obtained by computing the density within a small region around a given point of interest. The results strongly depend on the size of this region: the larger it is chosen, the smoother the appearance of the density estimate. For an “unbiased” determination of this size, use was made of the function bw.ppl in the R spatstat package (https://cran.r-project.org/web/packages/spatstat/index.html) which uses a cross-validation algorithm. This function suggested a smoothing bandwidth of several hundred units (more precisely: σ = 294, 556, 343, 415, 324 for human sections H11 blocks RO2 and RO4, chimpanzee, gorilla and guinea pig, respectively, with σ the standard deviation of an isotropic Gaussian smoothing kernel).

The K function (Ripley, 1987) was used to investigate the presence of local regularity within the area immediately surrounding a single bundle. The function K(r) describes (up to a scaling factor) the average number of points within a distance r around a point in the sample. For a homogenous, completely random (i.e., Poisson) process of density λ, there are λA points on average in a region of area A; the K function of this “benchmark” process is given by K(r) = πr^2^ and λK(r) yields the mean number of points. If the actually observed K function tends to be lower than this benchmark, then this behavior is interpreted as an indication of regularity because points tend to have fewer neighbors than expected from a Poisson process. Conversely, a larger K suggests clustering. In order to compare different sections, plots were made to show λK (i.e., the mean number of points within a circle of given radius) as a function of the scaled radius r^*^sqrt(λ). For a scaled radius of r^*^sqrt(λ)=1, λK = π for the Poisson process. In this study, λ was computed as the number of points in the analysis window divided by the window area.

## Results

### Pattern of myelin staining in transverse sections

**Figure 2:**
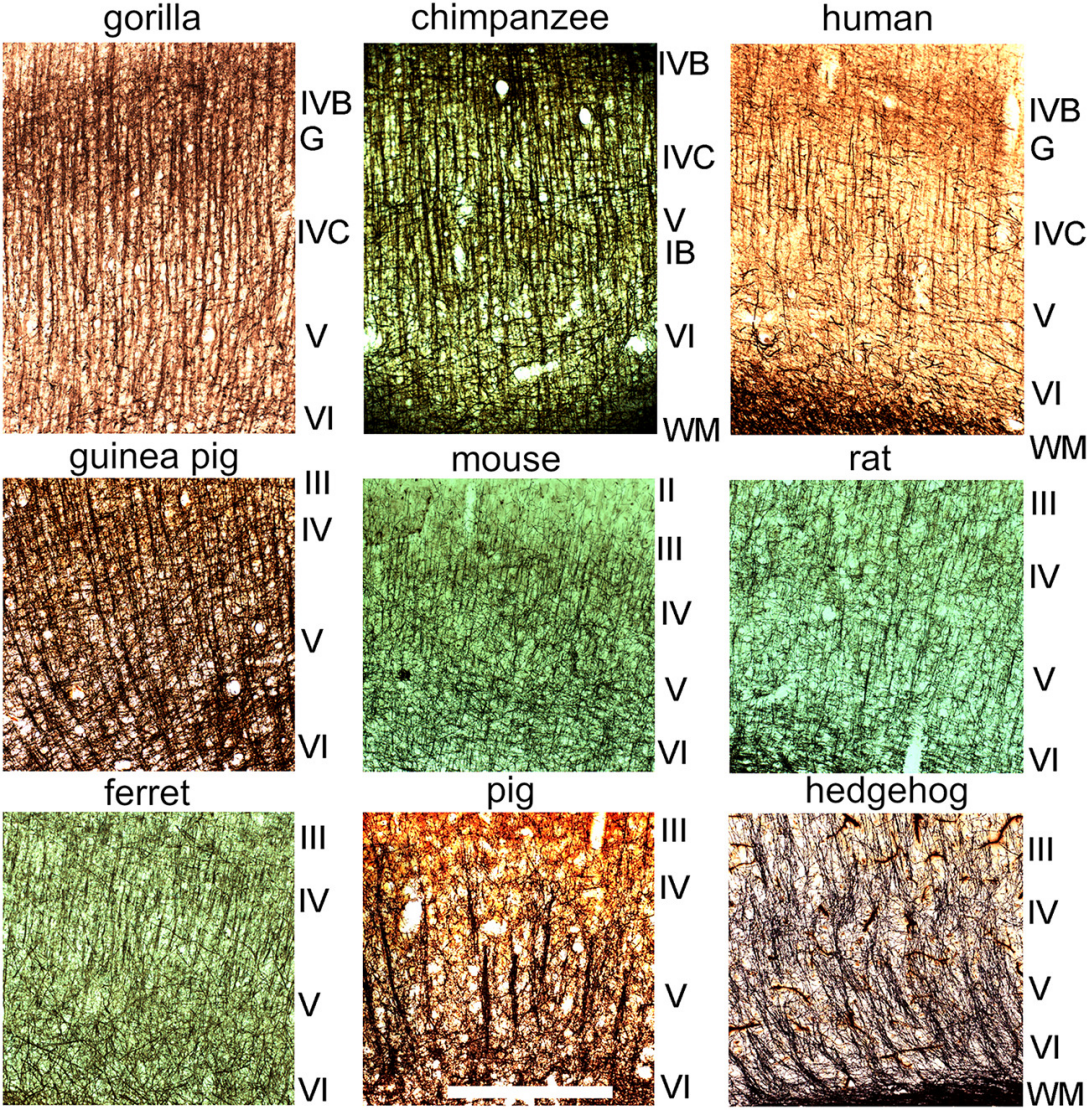
Sections cut in a plane orthogonal to the cortical surface to show myelin staining in layers II–VI of the primary visual cortex in nine different mammalian species. The layers are indicated by the Roman numerals to the right of each panel. The sections are shown at the same magnification and the scale bar is 500 μm. The stria of Gennari (G) of the three ape brains corresponds approximately to layer IVB while the inner band of Baillarger (IB) is seen most clearly in the chimpanzee brain and occurs within layer V.

Myelin staining was studied in transverse sections of V-1 in all nine species (Figure 2). Care was taken to obtain sections that were orthogonal to the cortical surface so that they were in the plane of any axonal bundles. Blocks were removed from the calcarine sulcus (when present) as well as the lateral surface of V-1 but their position was not exactly matched between species. The gorilla, chimp and human blocks all had very similar patterns of staining (Figures 1E, 2) with long, parallel bundles of myelinated fibers stretching from the base of layer III to the white matter. The bundles were spaced at fairly regular intervals and they produced a dominant radial pattern that was superimposed on the meshwork of stained fibers, many of which ran parallel to the surface especially in layer IVB (stria of Gennari). Similar prominent bundles of radially oriented fibers running from the base of layer III to the white matter with a regular spacing were also present in the guinea pig but were not as clear in the other species studied. In the ferret V-1 clear, regular bundles were present from the base of layer III into layer V but there was little evidence of bundles in layer VI where the background meshwork was more prominent. In the mouse there were thin, radial bundles from the base of layer III into layer V but in places they were either obscured or not present, and were even less evident in layer VI. In the rat there were short segments of myelinated bundles in layers III–VI but they were not as regular as in the guinea pig and seldom traversed more than two layers. In the pig there were thick, prominent bundles in layers V and VI but they were more widely spaced and less regular than in the ape brains. Darkly stained, radial fibers were present in the hedgehog but they were diffusely arranged and tended to only coalesce into clear bundles just before entering the white matter.

### Variability of inter-bundle distance compared across seven species

**Figure 3:**
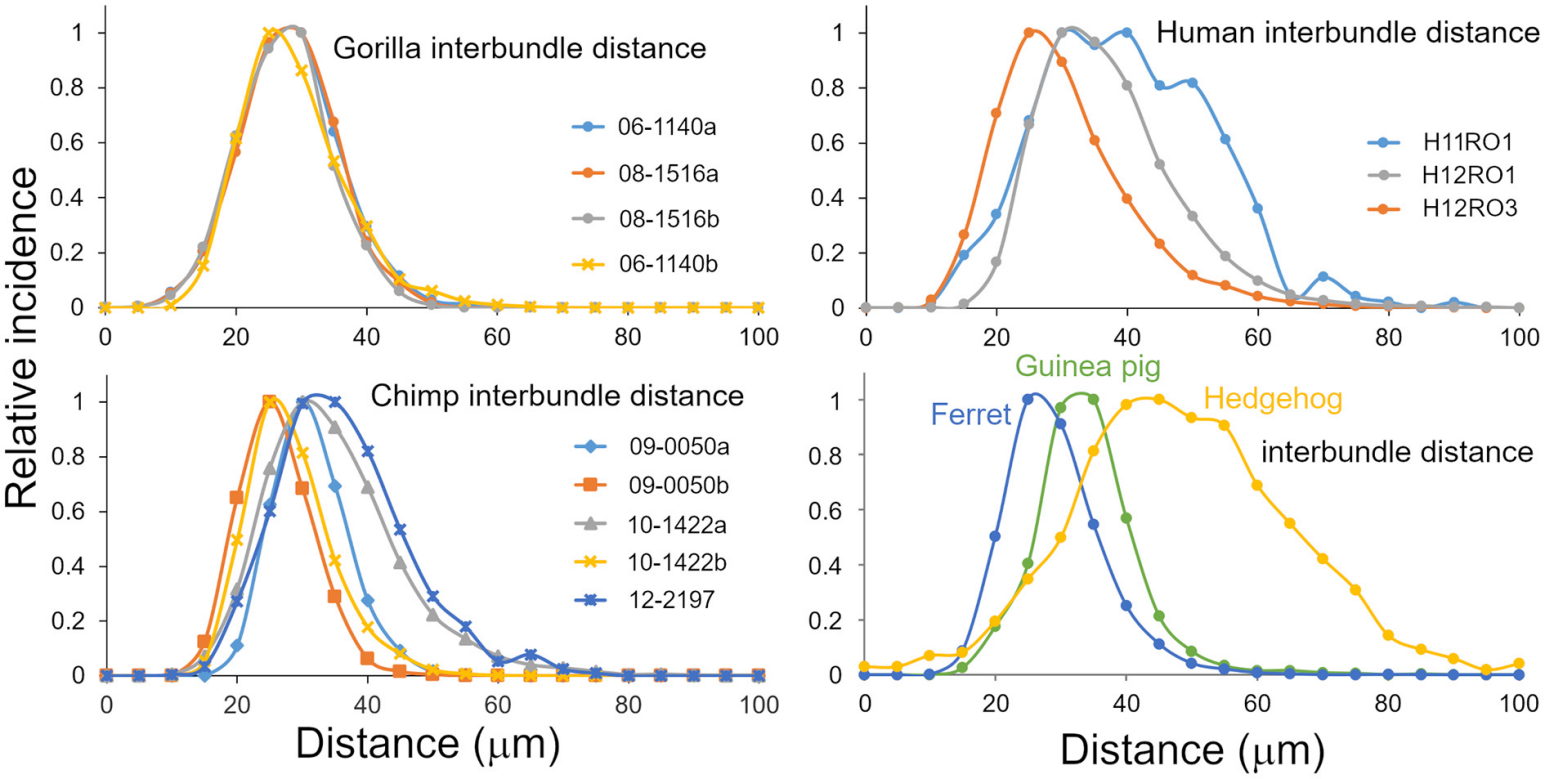
Distribution of inter-bundle distances measured in orthogonal sections from six species where there was a regular arrangement of fibers into bundles. Each plotted line is based on multiple sections from a different block as indicated by the legend.

**Figure 4:**
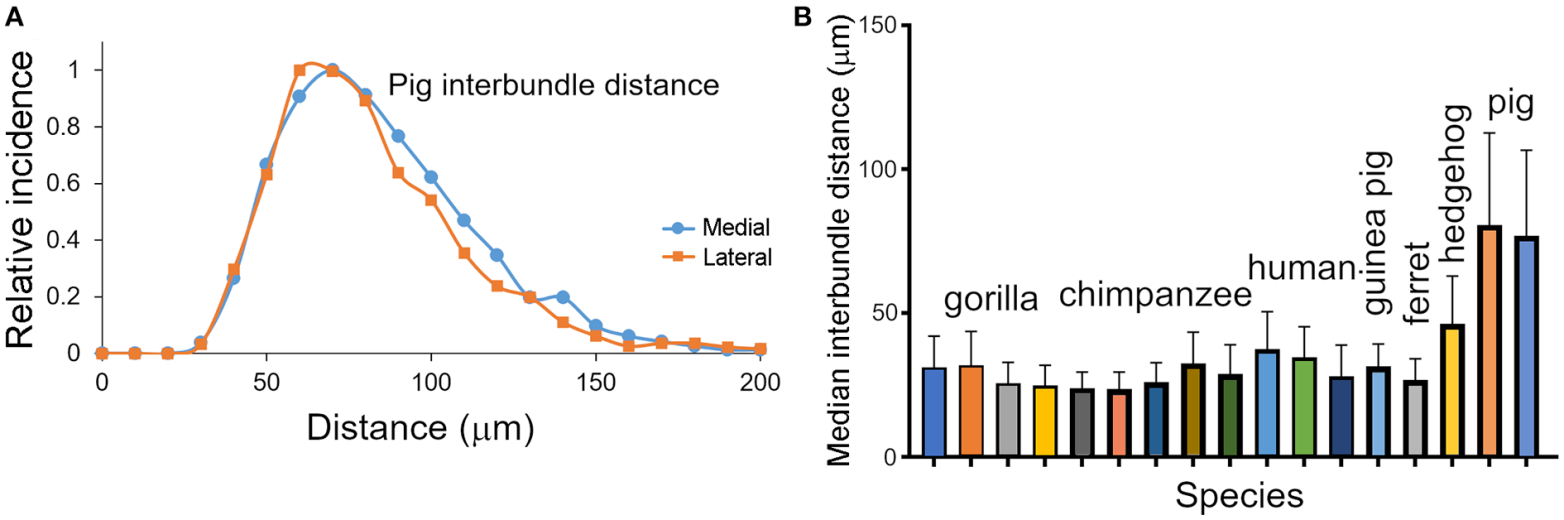
**(A)** Distribution of inter-bundle distances measured in orthogonal sections from two blocks of the pig brain. The distribution is flatter than the other species studied and has a different scale on the “X” axis. **(B)** Histogram showing the median values (with standard deviation) of the inter-bundle distance for orthogonal blocks from each of the species where counts were made. These are the same blocks as described in Table 1.

Inter-bundle distances were measured in blocks from seven species but not in the mouse or rat. For the sake of consistency measurements in orthogonal blocks were only made from the right hemisphere in each species. Although there was evidence of bundles in the mouse and rat area V-1, they were only present as short segments and it was decided that the material was not suitable for making accurate measurements. Measurements of inter-bundle distance were made in long segments of V-1 using the line measurement facility in the Neurolucida system. The distributions of inter-bundle distances were visualized by assigning each measurement to bins that incremented in 5 μm steps for 6 of the species (10 μm increments for the pig) and the normalized frequency plotted for each block (Figures 3, 4A). All the blocks had smooth, unimodal distributions but when they were tested for normality most had significant amounts of skew or kurtosis (Table 1). Only one gorilla and one human block had strictly normal distributions. The four gorilla blocks all had similar distributions but the five chimpanzee blocks showed clear differences from each other and this was also true of the three human blocks. The mean inter-bundle distances had similar ranges for five of the species: gorilla, 25–32 μm; chimpanzee, 23.7–32.6 μm; human, 28.1–37.6 μm; guinea pig, 31.5 μm; ferret, 26.8 μm. Thus, although the means for these five species were not the same, the variability between them was no greater than the variability between different blocks from the chimpanzee. Greater differences were found with the hedgehog brain (mean 46.4 μm) and particularly the pig brain which had, by far, the largest means (76.9 and 80.7 μm). The distances in the hedgehog brain were about twice the size of those in the ape brains while in the pig brain they were almost three times the size of those in the ape brains.

**Table 1:** Details of animals and analysis of inter-bundle distances where bundles were measured in orthogonal sections.

**Species**	**Gorilla**				**Chimpanzee**					**Human**			**Guinea pig**	**Ferret**	**Hedgehog**	**Pig**	
ID #	06-1140		08-1516		09-0050		10-1422		12-2197	11D25694	12D12468		GP1375	F32	HHGP1	abattoir	
Age	33 years		44 years		18 years		33 years		34 years	58 years	81 years		7 weeks	32 months	adult	6 months	
Weight (kg)	–		–		–		–		–	–	–		0.575	1.560	0.767	~70	
Sex	Male		Male		Female		Female		Male	Male	Male		Male	Male	Female	–	
Block	a	b	a	b	a	b	a	b		R01	R01	R03				Medial	Lat.
n (bundles)	1,876	1,719	2,330	2,534	5,353	5,052	6,996	5,016	1,009	318	4,868	5,291	1,404	3,769	2,242	2,066	1,887
Mean distance	31.1	32.0	25.7	25.0	23.9	23.7	32.6	26.1	28.9	37.6	34.7	28.1	31.5	26.8	46.4	80.7	76.9
Standard deviation	11.0	11.8	7.2	7	5.7	5.9	10.8	6.7	10.2	13	10.6	10.9	7.8	7.4	16.6	32	29.8
skewness	0.8	0.77	0.1	0.12	0.54	0.04	1.19	0.87	0.67	0.41	1.12	1.37	1.09	0.93	0.27	1.45	1.31
Kurtosis	0.92	0.78	0.1	0.06	0.45	0.11	3.14	1.36	0.57	0.17	2.42	3.83	3.73	1.78	0.24	4.54	2.72
Kolmogorov-Smirnov	0.07	0.06	0.02	0.02	0.04	0.01	0.07	0.07	0.07	0.04	0.07	0.08	0.06	0.06	0.03	0.08	0.09
*P*-value	0	0	0.11	0.04	0	0.04	0	0	0	>0.1	0	0	0	0	0	0	0

The different blocks were compared with non-parametric tests to quantify the differences in inter-bundle differences between different parts of V-1 from a single brain. The pair of blocks from gorilla brain 08_1516 were significantly different from each other (Mann-Whitney *p* = 0.0004) and while the other pair were not (Mann-Whitney *p* = 0.05), overall there was a highly significant difference between the four gorilla blocks in a one-way ANOVA (Kruskal-Wallis *p* < 0.0001). The pair of blocks from chimp brain 10-1422 were significantly different from each other (Mann-Whitney *p* < 0.0001) and while the other pair were not (Mann-Whitney *p* = 0.069), overall there was a highly significant difference between the five chimp blocks in a one-way ANOVA (Kruskal-Wallis *p* < 0.0001). The pair of blocks from human brain H2 were significantly different from each other (Mann-Whitney *p* < 0.0001) and from the block in brain H1 (Mann-Whitney *p* < 0.0001). In the four non-primate brains only the pig had two blocks stained from one brain but these were significantly different (Mann-Whitney *p* = 0.0001. Overall the four non-primate brains were different from each other (Kruskal-Wallis *p* < 0.0001). However, in ome cases the differences between two blocks from one brain were greater than between blocks from different species. This was seen most clearly in the case of the guinea pig brain which was not significantly different from the gorilla block 06-1140b where the medians were 30.9 and 30.4, respectively (Mann-Whitney *p* = 0.27). The guinea pig block was also not significantly different from the chimp block 10-1422a where the medians were 30.9 and 31.1, respectively (Mann-Whitney *p* = 0.19). These differences, between species, are shown graphically by plotting the median values (with error bars showing the standard deviations) in Figure 4B.

### Correlation between inter-bundle distance and other evolutionary factors

**Figure 5:**
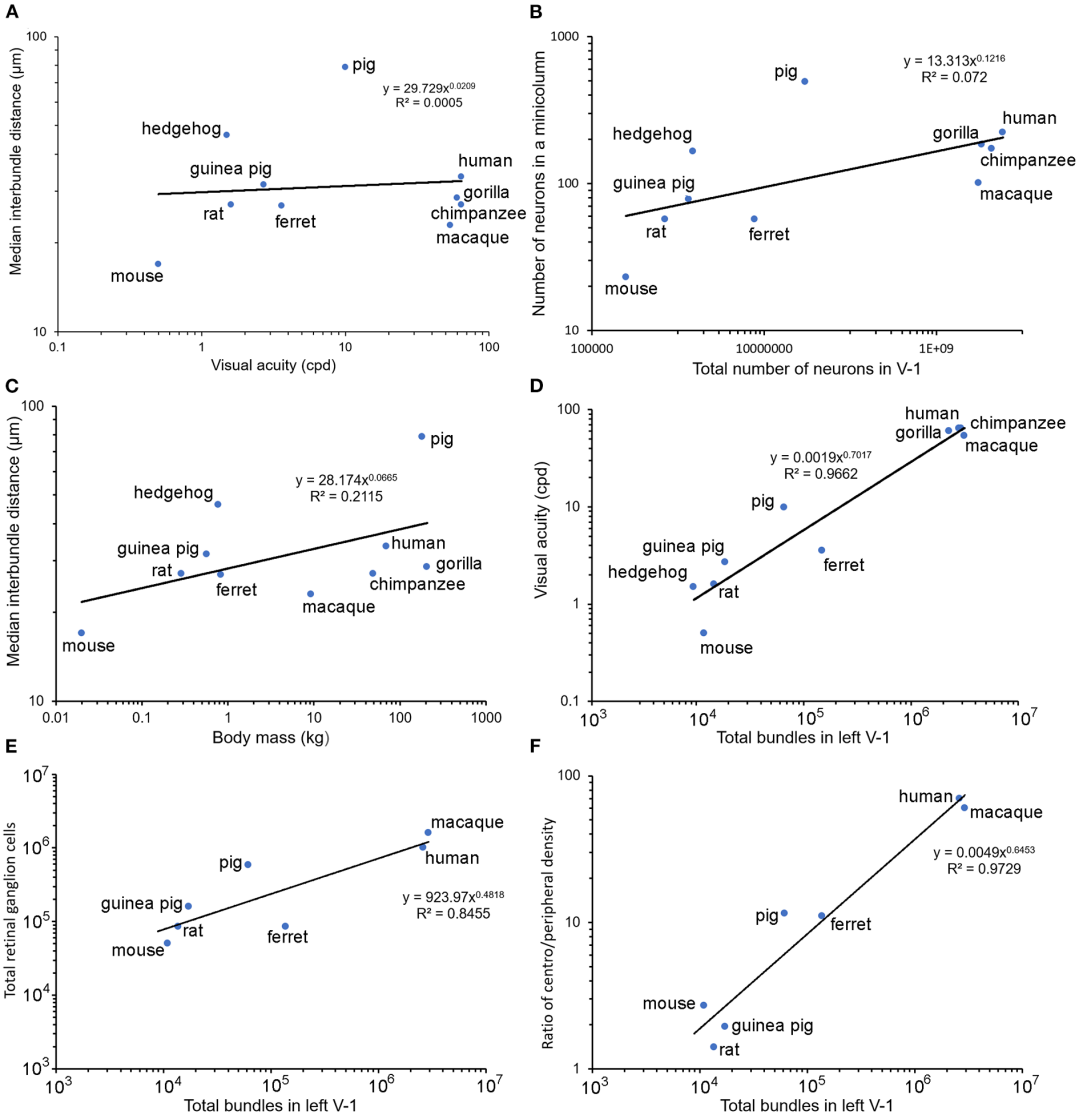
Pairs of relevant parameters are plotted against each other on log/log axes for each of the seven species used in the current study where available, along with values for the macaque monkey, rat and mouse published previously by others. Panel **(A)** shows a lack of any clear relationship between visual acuity (cycles per degree) and the median interbundle distance. Panel **(B)** also shows a lack of any overall correlation between the estimated total number of neurons in V-1 and the number within a single minicolumn. There is only a weak correlation between body mass and median interbundle distance **(C)**. However, when the total number of bundles in the left V-1 is plotted against visual acuity **(D)** there is a strong correlation overall (*R*^2^ = 0.97). A similar tight relationship is shown between the total number of retinal ganglion cells and myelin bundles in V-1 **(E)**. An even better correlation is seen when the ratio of the density of ganglion cells in the fovea/area centralis to the peripheral retinal density is plotted against myelin bundles **(F)**.

There were large differences between the inter-bundle distances for the pig and hedgehog and the other species. It is not clear what the reason might be for these differences but given that the different orders within the clade of placental mammals formed just after the Cretaceous-Paleogene boundary about 65 million years ago (O'Leary et al., 2013) it is likely due to differences that occurred during their separate evolutionary paths. Three factors that were considered to possibly have similar trends to inter-bundle distance during evolution were (1) visual acuity along with the related measurements of axial diameter of the eye and the total number of retinal ganglion cells, (2) degree of encephalization as measured by the surface area and total number of neurons in V-1, and (3) differences in body mass. The values for these factors, where available, are shown in Table 2 along with an estimate of the total number of bundles within the left V-1 based on bundle density. An estimate of the number of neurons in each mini-/microcolumn is also provided by assuming that each myelin bundle represents a cylinder with a diameter equivalent to the median interbundle distance and calculating the number of neurons based on their density under 1 mm^2^ of V-1. The relationship between (1) inter-bundle distance and visual acuity is plotted in Figure 5A, (2) the number of neurons in a minicolumn and the total number of neurons in V-1 is plotted in Figure 5B and (3) inter-bundle distance and body mass is plotted in Figure 5C. In each instance there is a large range of values and so they were plotted on a log/log scale. A regression line was calculated for each by fitting the data to a line based on a power relationship. There was no significant correlation overall between median interbundle distance and visual acuity (*R*^2^ = 0.0005) or between the median number of neurons in a minicolumn and the total number of neurons in V-1 (*R*^2^ = 0.072). There is a better overall correlation between mean interbundle distance and body mass (*R*^2^ = 0.21) but it is still quite weak. There may be a significant correlation between these factors when values are compared within one mammalian order but we didn't have measurements from enough species from within a single order to make a meaningful comparison.

**Table 2:** Visual and body measurements for the species used in this study compared to the macaque monkey.

**Order and species**	**AD eye^a^ (mm)**	**Total retinal GC^b^ (× 10^6^)**	**Visual acuity^c^ (cpd)**	**Body mass^d^ (kg)**	**V-1 area left (mm^2^)**	**V-1 total cell (*n*)^e^ × 10^6^**	**Inter-bundle distance (μm)**	**Bundle density (bundles/mm** ^**2**^ **)**		**Total bundles in left V-1**	**Total neurons in mini-column**
								**Orthog-onal**	**Tangen-tial**		
**Artiodactyla**											
*Sus scrofa*	24.8	0.584^f^	9.92	182	300^g^	30	**78.8**	**205**		61,500	490
**Eulipotyphla**											
*Erinaceus europaeus*	7.2	–	~1.5	**0.77**	15^h^	1.5	**46.4**	**591**		8,665	166
**Carnivora**
*Mustela putorius*	7.5	0.085^i^	3.57	**0.83**	78^j^	7.8	**26.8**	**1,773**		138,294	57
**Rodentia**											
*Mus musculus*	5.28	0.05	0.5	0.03	2.5^e^	0.25	17	4,406		11,015	23
*Rattus norvegicus*	5.58	0.086	1.6	0.29	7.1^e^	0.71	27	1,929		13,696	57
*Cavia porcellus*	8.7^k^	0.159	2.7^l^	0.57	**13.5**	1.35	**31.5**	**1,283**	**643**	**17,320**	**78**
**Primate**											
*Macaca mulatta^*PS*^*	20	1.6	53.6	9.25	1,269^e^	3,084	23^m^		2,318^m^	2,941,542	101
*Gorilla gorilla*	22.5	–	60	207	1,350^n^	3,375	**28.5**	**1,568**	**1,287**	**1,737,450** −**2,116,800**	**184**
*Pan troglodytes*	20.9	–	64.28	49.2	1,570^o^	4,380^p^	**27**	**1,746**	**1,243**	**1,951,510** −**2,741,220**	**172**
*Homo sapiens*	24.5	1	64	72	2,300^e^	5,957^p^	**33.5**	**1,135**	**368**	**846,400** −**2,610,500**	**222**

Bold figures were measured in the current study. Other values were obtained from published data as indicated by the superscript numbers. Column titles are abbreviated as follows: AD, axial diameter; retinal GC, retinal ganglion cells (in millions). Visual acuity was measured in cycles per degree (cpd).

a Howland et al., 2004;

b Baden et al., 2020;

c Veilleux and Kirk, 2014;

d Boddy et al., 2012;

e Srinivasan et al., 2015;

f (Garca et al., 2005;

g Fang et al., 2006;

h Kaas et al., 1970;

i Henderson, 1985;

j Law et al., 1988;

k Zhou et al., 2006;

l Bowrey et al., 2015;

m Peters and Sethares, 1996;

n de Sousa et al., 2010;

o Miller et al., 2014;

p Rockel et al., 1980).

There was a large range of inter-bundle distances between species within this sample of land mammals (17–79 μm), even when measured in a single cortical area (V-1). This corresponds to a large range in the number of neurons associated with each mini-/ microcolumn from 23 in a mouse microcolumn to 490 in a pig minicolumn (Table 2). The primates had intermediate values for neuronal number but still had a 2-fold range from 101 (macaque) to 222 (human). However, this variation is small compared to the total number of myelin bundles (minicolumns) within V-1 of different species, which ranges from about 8,665 in a hedgehog to over two million in large primates. Thus, a better correlation with visual acuity, across all mammals measured, was found when it was plotted against the estimated total number of mini-/microcolumns (based on the assumption that a single bundle represents a single minicolumn) as shown for the left V-1 (Figure 5D). There was a tight correlation (*R*^2^ = 0.97) between visual acuity and the total number of mini-/microcolumns. Visual acuity is thought to be tightly correlated with the number of retinal ganglion cells (Baden et al., 2020) and we confirmed that there was a tight relationship between the numbers of retinal ganglion cells and the total number of bundles in V-1 by plotting them together in Figure 5E. An interdependence between the numbers of retinal ganglion cells and the total area of V-1 has already been shown in the human (Andrews et al., 1997) but recent work has suggested that a better indicator of V-1 area and functional complexity is the central-to-peripheral ratio (CP ratio) of retinal cell density (Ibbotson and Jung, 2020). Values for this ratio have been published for many mammals and these were used to show a high correlation between the CP ratio and the total number of bundles in V-1 (Figure 5F).

### Changes in inter-bundle distance within a single cortical block

**Figure 6:**
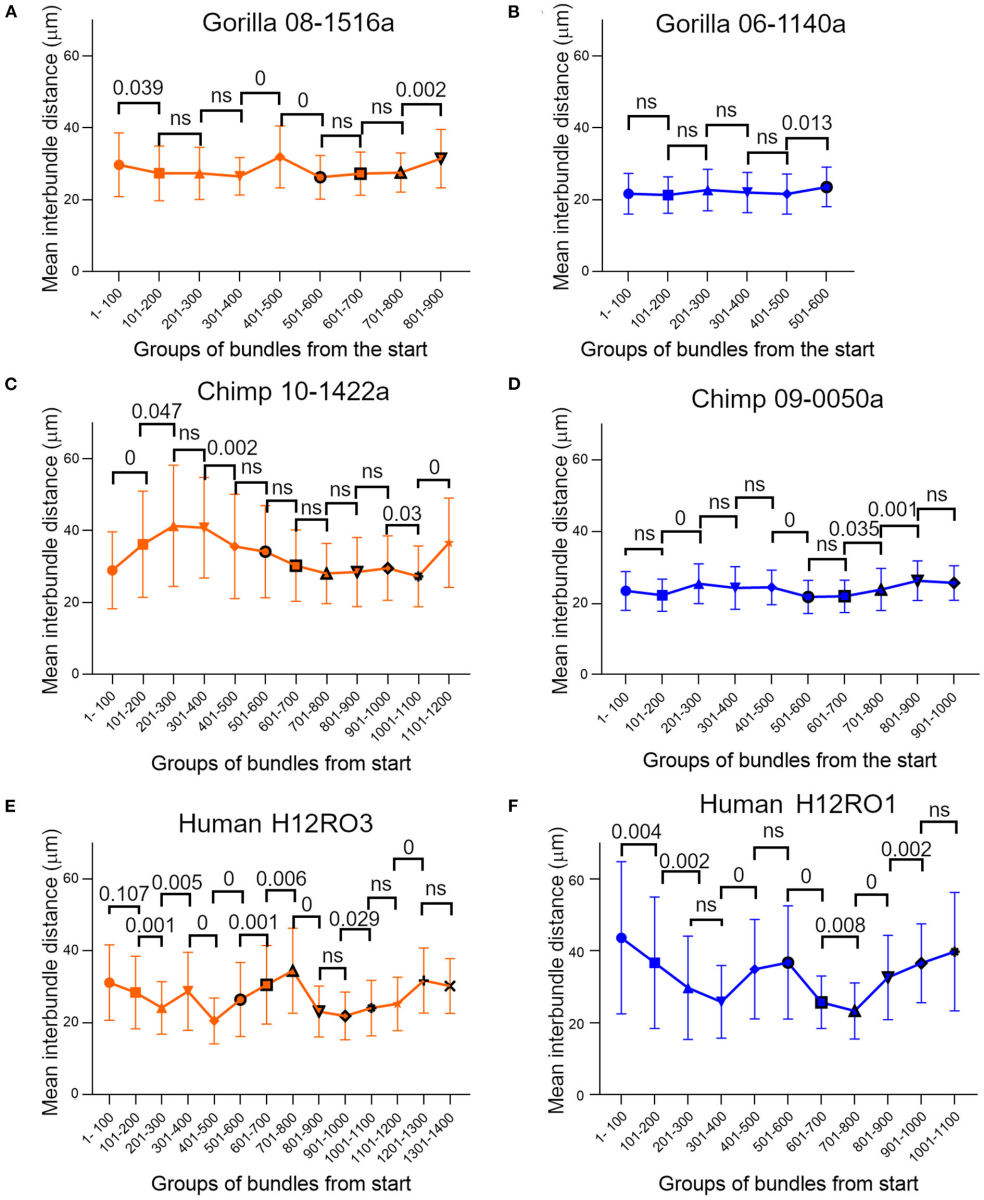
Plots of mean inter-bundle values based on sequential groups of 100 bundles measured in layer V of orthogonal blocks from human and ape brains where there was at least 10 mm of cortical primary visual area containing clear bundles. The error bars indicate the standard deviation for each group. For all three species the mean values were not constant but varied across the cortical surface. Most groups of bundles were not normally distributed and in order to compare the difference between consecutive groups statistically, the median values were compared with a Mann-Whitney test and the probability value plotted above the pairs of means in panels **(A, C–F)**. For some pairings the medians show no significant (ns) difference at the *p* = 0.05 level. In panel **(B)** all the groups of bundles had normally distributed values and so the difference between their sequential mean values were compared with an unpaired *t*-test.

There was a range of inter-bundle distances within any one cortical block, but this variation was not arranged randomly. Instead, there were consistent gradients of spacing within each block so that the density of bundles could be significantly different from one part to another. To demonstrate this, we chose two blocks from V-1 of each of the great apes where there was at least 10 mm of cortical tissue with an uninterrupted stretch of well-stained bundles. The mean inter-bundle distance of sequential groups of 100 of these bundles were plotted along with their standard deviation and are shown in Figure 6. The distribution of distances within each group of bundles was usually not normally distributed. Therefore, to determine if adjacent groups were significantly different from each other, the median values were compared with a Mann-Whitney test and the probability value plotted above the pairs of means in Figures 6A,C–F. In one of the gorilla blocks all of the 100 bundle groups were normally distributed and so the means were compared with an unpaired *t*-test. Some pairs of groups showed no significant difference, but others showed highly significant differences. In some stretches the mean distances changed in a consistent direction over three or even four groups and implied that there were significant changes in the density of bundles within V-1 from a single specimen for each of the three species.

### Distribution of myelinated bundles in tangential sections

**Figure 7:**
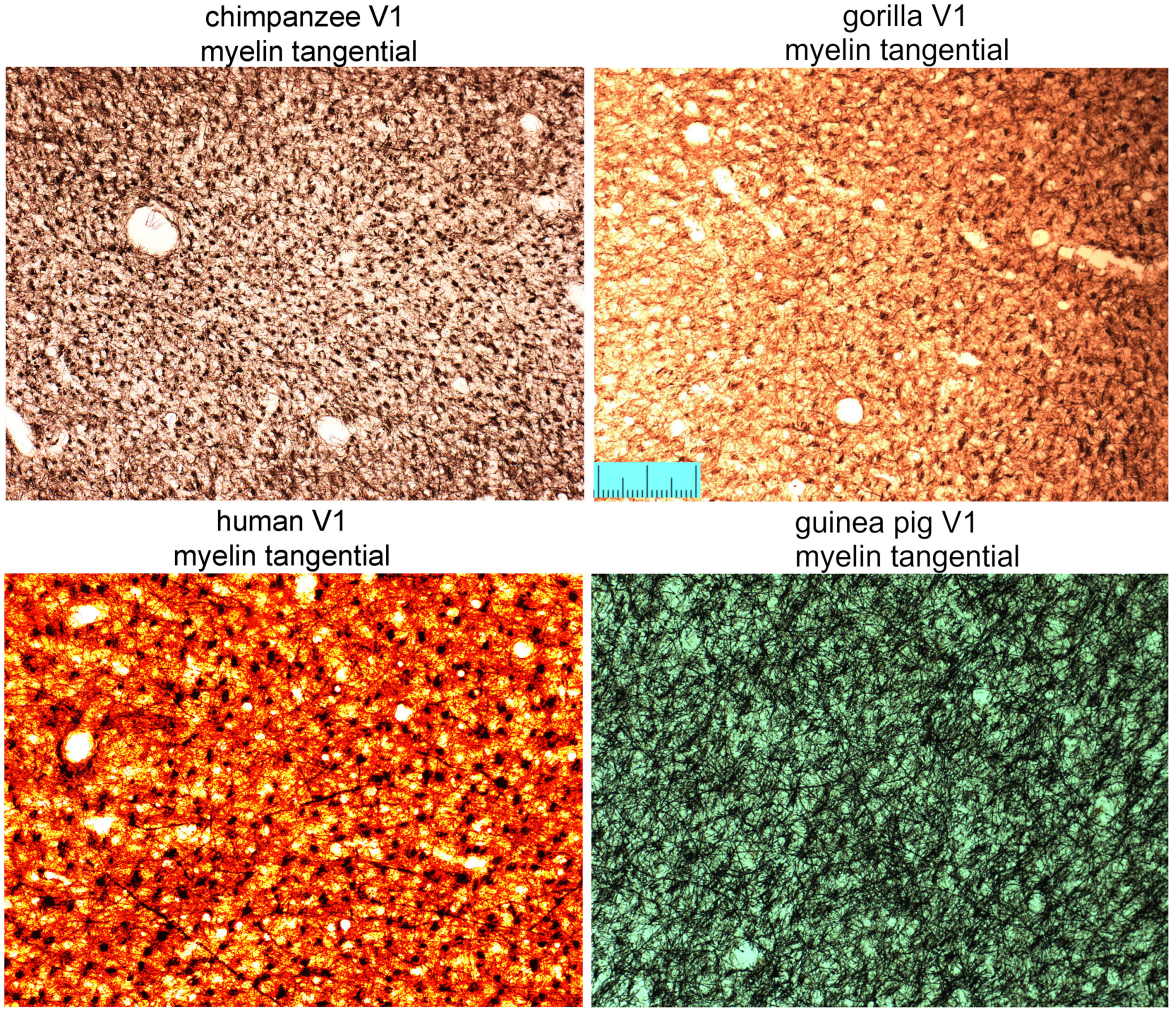
Tangential sections through layer V of the primary visual area (V1) stained for myelin to show the regularity of bundles of descending fibers in three great apes and the guinea pig. Scale bar is 200 μm.

**Figure 8:**
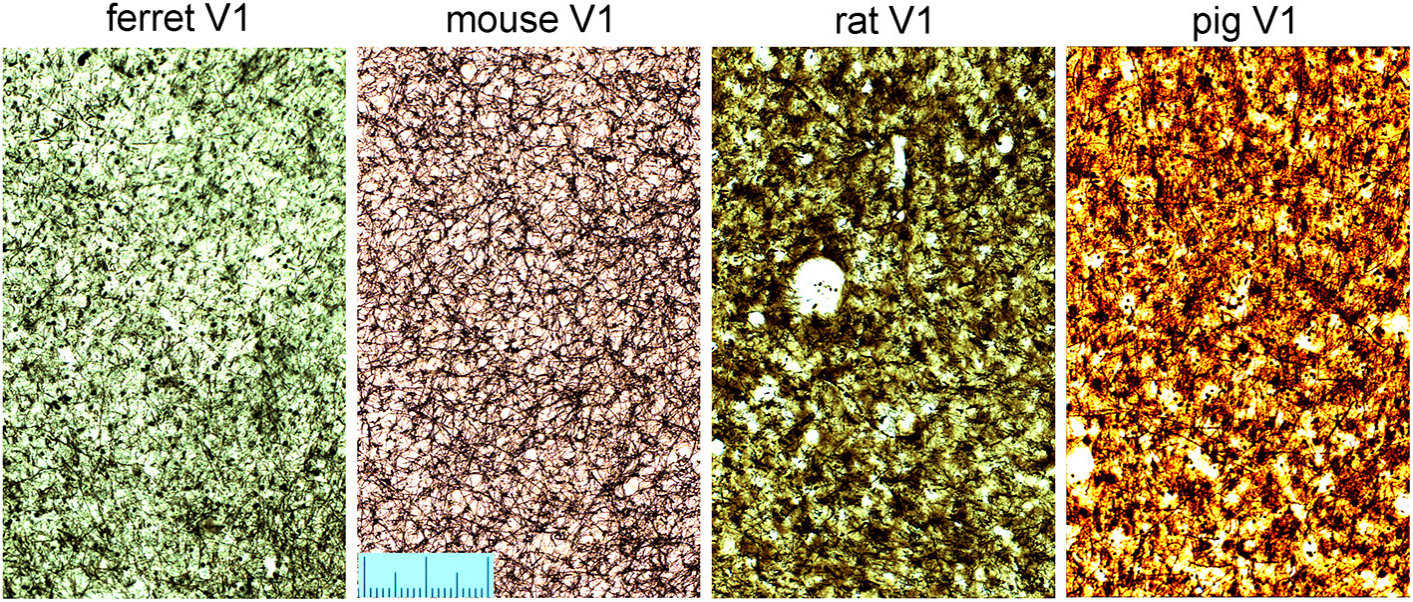
Tangential sections through layer V of the primary visual area (V1) stained for myelin to show the pattern of myelinated fibers in four other mammalian species. The scale is 200 μm long.

The density of bundles was studied directly by viewing the bundles cut *en face*. As the primate blocks were all taken from archival material no attempt was made to flatten the cortex from them or the other gyrencephalic brains before sectioning. Only samples of rodent brain were gently flattened during immersion fixation to increase the area of tangential sections and help with identifying the borders of V-1 (Figure 1D). All three ape brains had discrete puncta of myelin staining that represented the myelin bundles (Figure 7). The puncta did not have a strict geometric arrangement but were fairly regularly spaced apart from when their pattern was disrupted by the presence of a blood vessel. When guinea pig sections were compared (Figure 7), there were also puncta of myelinated fibers but they were less discrete than in the apes. The guinea pig puncta were less compact and there were more myelinated fibers running between the bundles. The ferret bundles were smaller and more compact than the guinea pig while the mouse, rat and pig sections had many myelinated fibers running within the plane of the section so that the bundles seemed to be more like the nodes of a meshwork rather than discrete bundles (Figure 8). Only one brain was available for the hedgehog and it was not sectioned in the tangential plane.

The density and regularity of the myelinated bundles was measured by plotting the centers of the bundles using the Neurolucida reconstruction software so that the bundle density could be measured directly in tangential sections. The bundle densities for the chimpanzee and the gorilla are almost equal (1,243 and 1,287 bundles/mm^2^ respectively) and about a factor of three larger than in the human samples (section H11RO4: 314 and section H11RO2: 422 bundles/mm^2^). The density for the guinea pig has an intermediate value (643 bundles/mm^2^).

### Macro inhomogeneity of bundles across the whole extent of tangential sections

**Figure 9:**
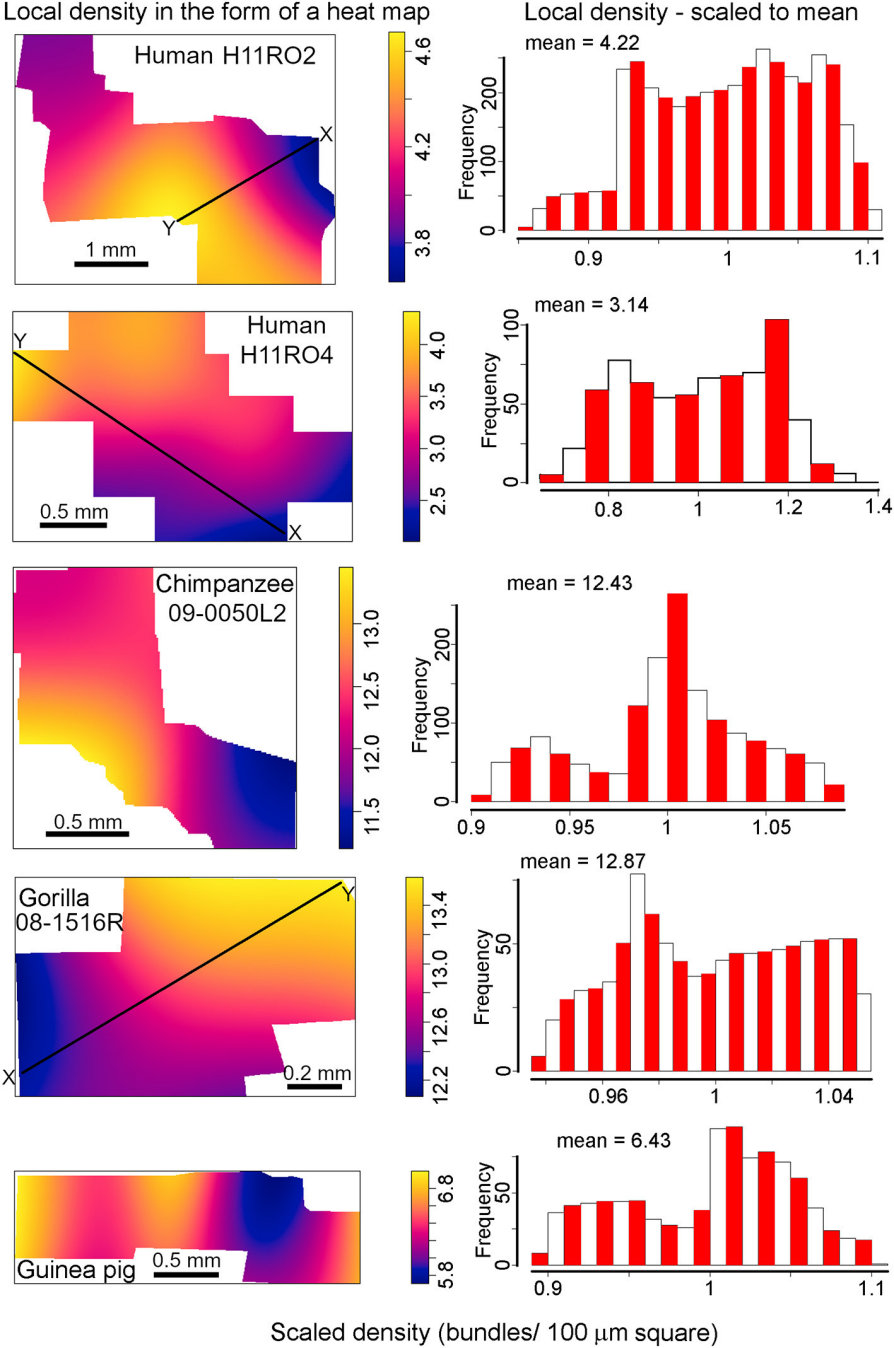
Digital reconstructions of myelinated bundles in tangential sections through layers IVC or VA from the four species with the most regular bundles. The density of the bundles is displayed as a heat map. Beside each heat map is a histogram showing the distribution of density values for bundles in the section relative to the mean density which was normalized to represent 1. The mean value above each histogram gives the density in terms of bundles within a square with a side of 100 μm. These values have to be multiplied by 100 to give the density in mm^−2^. The black lines labeled “X to Y” indicate monotonic variations in density which are given numerical values in the text.

The plots in Figure 9 show “heat maps” of the densities obtained using the R spatstat package as well as histograms of the densities (scaled to their means) which allowed for a better representation of the range of inhomogeneity. These results indicate that all samples show some degree of inhomogeneity, but it is especially strongly pronounced in the human sample of section H11RO4. In this section there is a doubling of local density along a line of distance 2.5 mm (between points “X” and “Y”) from 204 to 424 bundles/mm^2^ while in section H11RO2 along a line of similar length (2.25 mm from “X” to “Y”) the density changes from 360 to 464 bundles/mm^2^. The gorilla sample is the most homogeneous and along a line marking the steepest gradient of change (1.4 mm from “X” to “Y”) the density only changes from 1,210 to 1,364 bundles/mm^2^. The guinea pig density is more structured than the others (two “valleys”) but like the gorilla it has a low range of values (579–707 bundles/mm^2^) while the chimpanzee block also had a low range of values (1,120–1,367 bundles/mm^2^).

### Regularity of bundle position relative to its nearest neighbors

**Figure 10:**
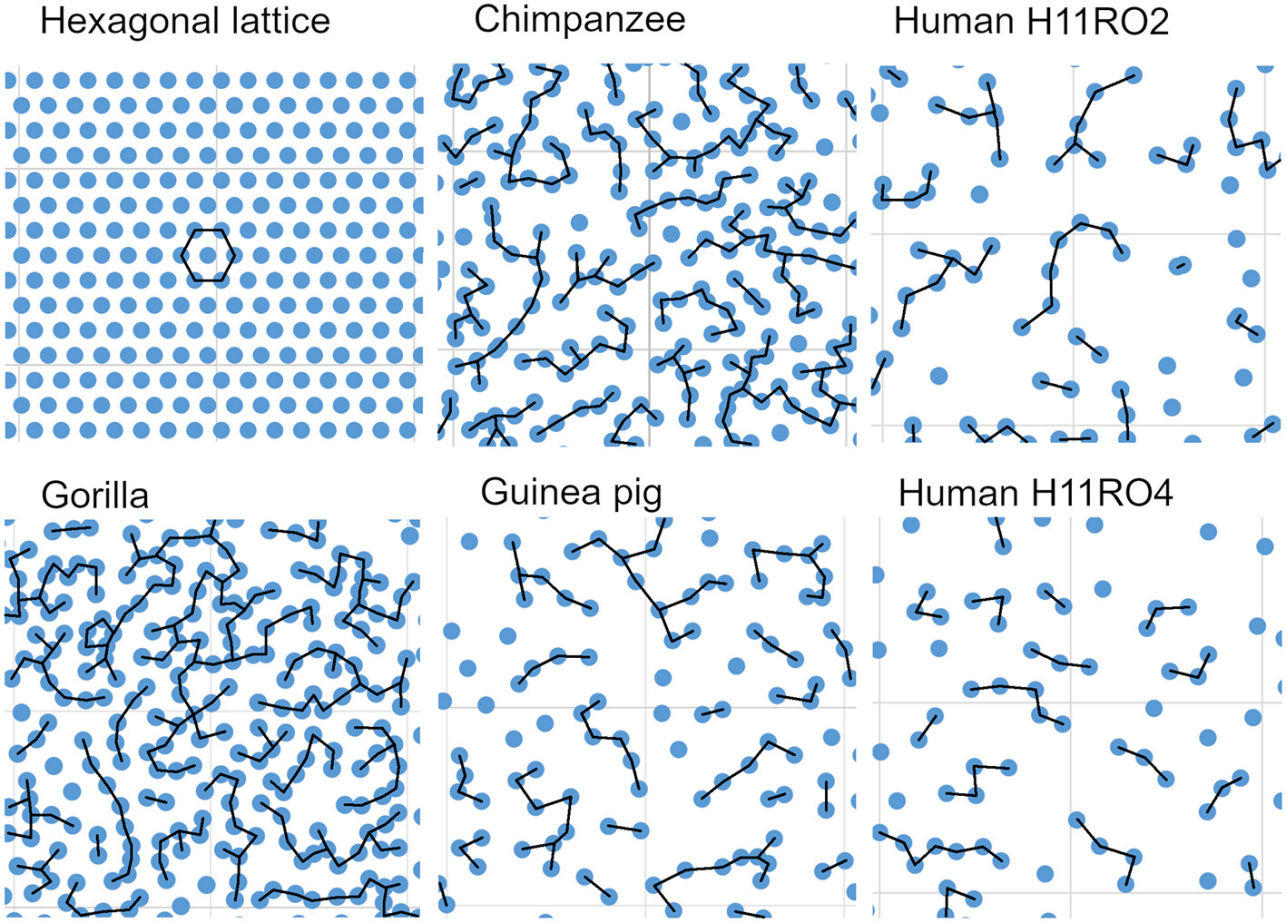
Comparison of an imaginary hexagonal lattice, with the arrangement of myelin bundles from representative areas from the five sections that were digitally reconstructed. In each case a bundle is represented by a blue circle (diameter 17 μm). In the hexagonal lattice each “bundle” is equidistant from its six nearest neighbors (26.67 μm) while in the cortex the bundles form branched strings of closely adjacent bundles the centers of which are usually <27 μm apart from their nearest neighbor (as indicated by the black lines). Each panel has a background lattice with a thin gray line indicating squares with a side of 200 μm.

The simplest arrangement for orientation columns in V-1 would be to have a hexagonal arrangement (Grabska-Barwinska and von der Malsburg, 2008) and modeling studies have suggested that the anatomical modules may also be arranged in a hexagonal lattice as indicated in the first panel of Figure 10. In this configuration each bundle is equidistant from its six closest neighbors. In the tangential sections of this study this regular geometric arrangement was not observed. Instead, the bundles were arranged in linear or branched strings of closely adjacent bundles that were separated by broader bands where there was an absence of bundles. This is illustrated by the other panels in Figure 10 which show parts of each reconstruction made from a section through the cortex near the layer IV/V boundary in the three great apes and guinea pig. Each bundle is represented by a blue circle (diameter 17 μm) and the black lines indicate the position of the nearest neighbors.

**Figure 11:**
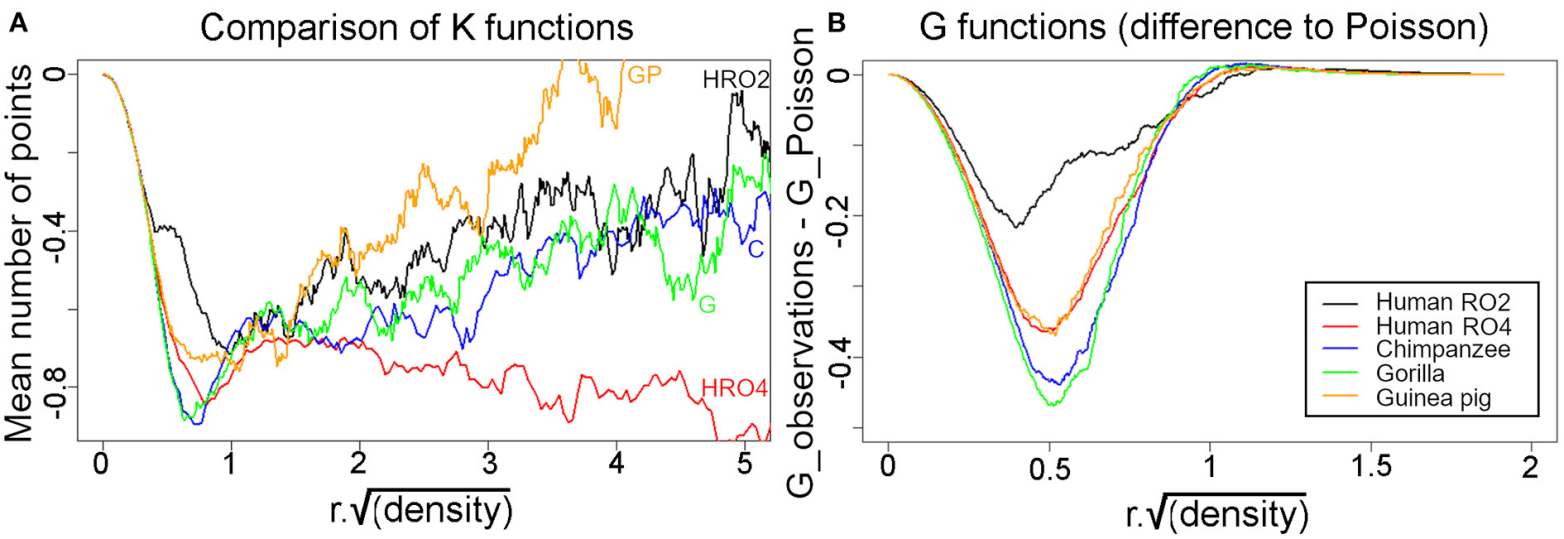
**(A)** Superimposed K functions from the five different tangential sections were superimposed for comparison and all show that the mean distance between a bundle and its nearest neighbors is always less than would be expected if they were organized at random. The only exception is when distances are measured to larger numbers of neighbors that are further away in the guinea pig, when they start to become more random. **(B)** Plots of the G functions measured as the difference from a Poisson distribution for each of the five sections. In each case when the nearest neighbors are considered they are further away from the central bundle than would be expected for a random arrangement.

To study the nearest neighbor relationships more rigorously for the four species where reconstructions were made from tangential sections, K functions were calculated and superimposed in Figure 11A. The graphs show that after accounting for the differences in packing density (λ), the K functions look remarkably similar. Most importantly, all K functions are negative [apart from the guinea pig for r^*^sqrt(λ) > 4], i.e., they indicate some degree of regularity in the arrangement of the bundles. Furthermore, they show that for small r^*^sqrt(λ), the decrease in K compared to the Poisson process is quite appreciable. For example, for r^*^sqrt(λ) ≈ 1, the difference is around 0.7. This means that in the Poisson case there will be around π≈3.14 points on average in a circle of radius r^*^sqrt(λ) = 1 around a given point, while in our data samples there are only 3.14–0.7 = 2.44 points. For larger r^*^sqrt(λ), the difference in K for the human section H11RO4 sample continues to decrease whereas in the other cases, it increases again toward 0. However, at these larger radii the differences are small relative to the total number of points; for example, a circle with radius r^*^sqrt(λ) = 5 contains about π^*^5^2^ ≈ 79 points in the Poisson case, and even for human section H11RO4 there is only about one point less!

Another way of looking at local regularity in the pattern of axon bundles is the nearest-neighbor (Clark and Evans, 1954) test. It compares the observed mean nearest-neighbor distance to the Poisson case. A larger mean distance is again taken as evidence for regularity while a shorter mean distance indicates clustering. The implementation of this test in the R spatstat package assumes homogeneity and except perhaps for the human section H11RO2, the local inhomogeneity is sufficiently mild that the test is still safely applicable. The results for the ratios are: human section RO2: R = 1.20, human section RO4: R = 1.35, chimpanzee: R = 1.41, gorilla: R = 1.41, guinea pig: R = 1.35. Again, these ratios are remarkably similar to each other (except perhaps Human section RO2 for which R is somewhat smaller, but this sample is also most strongly affected by inhomogeneity). The *p*-values for the test against a Poisson process are always around 0.002, i.e., there is highly significant evidence for regularity.

The nearest-neighbor distance distribution function G(r) gives the proportion of nearest-neighbor distances below r. It is thus closely related to the Clark-Evans tests but provides more detailed insights. The plots in Figure 11B show the difference between the observed values and the Poisson case. As expected, the G functions were quite similar apart from human section RO2 which deviated less from the Poisson process than the rest. These plots again indicate the presence of regularity in the bundle spacing.

## Discussion

### Size and density of fiber bundles across mammalian orders

The main aim of this study was to confirm the presence of radial bundles of myelinated fibers in V-1, across examples taken from five mammalian orders. Use of the same silver staining method on immersion-fixed, postmortem tissue facilitated a direct comparison. We found that the length, compactness and regularity of the myelin bundles varied between species with the clearest, most regularly arranged bundles present in the three great apes. Well-defined and regular bundles were also found in the guinea pig and ferret. All the species examined showed evidence of bundles of myelinated fibers in V-1. Bundles in the hedgehog were the least compact and least regular while those in the mouse and rat were only present for short distances and did not travel all the way from the base of layer III to the white matter. Although the bundles in the pig were thick and compact, they were widely and irregularly spaced.

These differences could be related to the different phylogenetic histories of the species studied and an attempt was made to find a correlation between inter-bundle distance and other relevant variables. However, there was no overall correlation between inter-bundle distance and body mass or visual acuity (Figure 5). As the area of V-1 increased there tended to be an increase in the inter-bundle spacing with the largest spacing between bundles in the human. This relationship was also found for the cylinders of neuronal somata where there seems to have been an increase in the inter-column distance with increasing encephalization: the narrowest, most densely packed columns were in the monkey cortex and the most widely spaced columns were in the human (Buxhoeveden et al., 2001; Semendeferi et al., 2011; Buxhoeveden, 2012). However, the strongest correlation overall across mammals was the relationship between the total number of mini- or microcolumns in the V-1 and visual acuity. On this relationship the chimpanzee and human have very similar values because although the area of V-1 in the chimpanzee is smaller than the human, the chimpanzee bundles are more densely packed. It has already been suggested that visual acuity is related to the computational power of the brain because (a) diurnal primates have a much higher visual acuity than non-primates with a similar brain size because of the higher density of neurons in their V-1 (Srinivasan et al., 2015) and (b) the optical quality of the guinea pig eye is much higher than would be expected from their limited visual acuity (Singh et al., 2020). It is possible that the key feature in computational power may be the number of functional units (minicolumns) rather than the density of cells *per se* in the neocortex. Numbers of myelinated bundles (minicolumns) may be a better indicator of computational power than V-1 cell density.

### Relationship between bundles of myelinated fibers and functional minicolumns

The tight correlation between the total number of minicolumns and visual acuity led us on to consider the functional significance of the fiber bundles. The presence of clear bundles of myelinated fibers in many cortical areas of the human brain (bundles of Meynert) has long been known because of classical studies, especially the work of the Vogts on myeloarchitectonics (summarized by Nieuwenhuys, 2013). These radial fiber bundles were complimented by the presence of distinct cylinders of Nissl-stained cells which are particularly prominent in the young human brain (Conel and Le, 1967). The radial cylinders of neurons in the human V-1 have been shown to correspond with the bundles of myelinated fibers in a study using adjacent sections. The columns defined by the two methods were found to have the same width and appear to be different parts of the same structure (Casanova et al., 2008). Cortical modules in V-1 that are composed of vertical cylinders of cells tightly associated with bundles of myelinated fibers have also been described in the macaque VI (Peters and Sethares, 1996). These are mainly composed of descending efferent axons from radial cylinders of pyramidal cells and composed of 7–72 (mean 34) axons some of which are unmyelinated (Peters and Sethares, 1996). We have assumed that this tight coupling between pyramidal cells and the radial bundles of fibers is true in all the great apes and most primates. Other work also pointed to a uniform arrangement of repeating cellular cylinders (Buxhoeveden and Casanova, 2002b) associated with radial bundles of apical dendrites (Innocenti and Vercelli, 2010), bundles of myelinated output axons and bundles of intrinsic, inhibitory axons from double bouquet cells (DeFelipe et al., 2006). All four types of cellular element: somata, apical dendrites, efferent (excitatory) axons and inhibitory intrinsic axons are now thought to be associated with the same cortical modules (Peters and Sethares, 1996; Jones, 2000; Buxhoeveden, 2012). These anatomical descriptions complimented the ideas of Mountcastle (1978) who defined the minicolumn as the basic modular unit of the neocortex and suggested that the human neocortex might contain as many as 600 million of them.

Rockland and Ichinohe (2004), like Mountcastle (1997), defined a cortical column as a group of interconnected neurons that share a certain set of properties and extend vertically through the cortical layers. Each column should have a common input, a common output and common response properties but in practice this has been difficult to establish even for an intensely studied area like V-1. Columns in V-1 have been shown to have a common input and common response properties, but it has been difficult to show that these types of columns also have a common efferent target. Indeed, most studies of neocortex would suggest that pyramidal cells in layers II/III project to other cortical areas while those in the deep layers project subcortically and so each minicolumn must project to at least two different targets. Much of the output from primate V-1 is to either V-1 or the second visual area (V2) (Gilbert and Wiesel, 1989; Schmidt et al., 1997) but V-1 also projects to other cortical areas (Rosa et al., 2009) and in rodents the majority of the outputs are subcortical (Hosoya, 2019). Primate V-1 is organized into a number of superimposed maps for features such as retinal position, orientation preference, ocular dominance, spatial frequency and the direction of motion (Hubel and Wiesel, 1977; Blasdel and Salama, 1986; Ibbotson and Jung, 2020) and so cellular cylinders will have a distinctive and common input. When line orientation preferences were examined in a single radial track through V-1 they were often found to have the same orientation for much of the track (Blasdel, 1992). These orientation preferences can show sudden discontinuities or rapid transitions over a distance of 40 μm in the tangential plane (Hubel and Wiesel, 1977; Blasdel and Salama, 1986) and it appears that a single orientation preference shown by a group of complex cells may be related to a single cylinder of neurons that all have the same preference (Mountcastle, 1997). However, the exact relationship between orientation columns and minicolumns has not been established. Orientation preference is organized into maps based on a pinwheel pattern (Blasdel and Salama, 1986; Hubener et al., 1997; Cloherty et al., 2016; Weigand et al., 2017) but the groups of cells with the same preference have different widths and it is clear that there are many more myelin bundles than pinwheels within V-1. The numbers of pinwheels in V-1 of various species has been estimated and varies from 429 in the ferret to 10,152 in the macaque monkey (Ibbotson and Jung, 2020). We estimated the number of myelin bundles in V-1 of these two species as being 138,294 and 2,941,542 respectively (Table 2). This would mean that each pinwheel would contain about 322 (ferret) or 290 (macaque) minicolumns. If you assume that each pinwheel has 15 orientation columns within it and that these radiate out from the center (Hubener et al., 1997) then each orientation column would contain about 20 minicolumns. Thus, a single minicolumn in the macaque monkey V-1 appears to have common inputs, common response properties and an interconnected set of vertically arranged neural elements that cross the full depth of the cortex. This may also be true of V-1 in all primates including the human.

Orientation columns have also been shown in the V-1 of carnivores such as the cat (Hubel and Wiesel, 1963; Hubener et al., 1997) and ferret (Kaschube et al., 2010) and there is also evidence of minicolumns in the cat V-1 based on radial bundles of myelinated axons (Hubel and Wiesel, 1965) and apical dendrites with a spacing of 56 μm (Peters and Yilmaz, 1993). It seems reasonable to suppose that the myelinated bundles in these species may also correspond to cellular modules with iso-orientation properties. The other species in our study with clear, regular fiber bundles is the guinea pig which is a hystricomorph rodent. There has been some preliminary evidence of orientation sensitivity in the guinea pig (Chebkasov, 1998) and another hystricomorph rodent (the red-rumped agouti) showed evidence of orientation selective modules that may extend across more than one layer (Ferreiro et al., 2021). However, there has not been any evidence found of orientation columns crossing all six layers in rodents, even in highly visual ones like the gray squirrel (Van Hooser et al., 2005; Laramee and Boire, 2014). The hystricomorph rodents have a separate evolutionary history from the rat-like (Myomorpha) and squirrel-like rodents (Sciuromorpha) which seem to have formed separate sub-orders about 65 million years ago (D'Erchia et al., 1996) and it is not too surprising that hystricomorph rodents may have a different organization to other rodents. Recent studies of the mouse V-1 have shown that cells with the same orientation preference in layers II and III are located in small modules of about 40 μm across and 120 μm deep (Ringach et al., 2016) and there are also bundles of layer V apical dendrites from putative minicolumns that show similar response properties (Kondo et al., 2016). Thus, there is evidence for clustering of cells with the same orientation preferences but no evidence that these form distinct columns stretching from layers II to VI. This ties in with our study of the myelinated bundles in the rat and mouse V-1 which showed short bundles of myelinated fibers that were mainly restricted to one layer. Studies of orientation preference in the V-1 of many species (Kaschube, 2014; Schmidt and Wolf, 2021) has led to the suggestion that there may be two types of organization: (1) an orientation preference map with smooth gradients of changing orientation preference centered on a pinwheel structure (primates and carnivores) forming a columnar arrangement extending through multiple cortical layers and (2) a “salt-and-pepper” arrangement that lack a smooth change in orientation preference and have an interspersed arrangement. The long radial bundles of myelinated fibers that extend down to the white matter in the guinea pig V-1 is a bit of an anomaly and suggests that some species may have clear minicolumns in V-1 that are not related to orientation columns. Other mammalian orders as represented by the pig and hedgehog appear to have a much less regular pattern of minicolumns and it looks unlikely that they will have a clear orientation preference map or even a regular pattern of minicolumns.

### Lack of uniformity of minicolumns between cortical areas and species

Initial studies of the uniformity of the neocortex indicated that the same number of neurons (80–120) can be found in 30 μm wide cylinders across several different cortical areas and species (Rockel et al., 1980; Carlo and Stevens, 2013). The main exception was that the primate V-1 had additional neurons in an expanded layer IV (270 neurons/cylinder). This led on to the hypothesis that there is a canonical minicolumn (Douglas et al., 1989) with a specified complement of different neuronal types and a standard set of intrinsic connections that together make up a narrow cylinder of cells that are vertically connected so that they have the same functional properties linked to a common set of inputs and outputs. However, more recent work has not supported the hypothesis of uniformity across the cortical sheet or the presence of canonical minicolumns (Beul and Hilgetag, 2014; Plebe, 2018). One way of demonstrating that there are functionally related changes between cortical areas has involved the analysis of the basal dendrites of layer III pyramidal cells in the macaque and marmoset monkeys. This has shown that the tangential area, complexity of the branching pattern and spinal density is low in V-1 and then increases successively in V2 and more rostral visual areas (Elston and Rosa, 1997, 1998; Elston et al., 1999). This work involved tangential slices and did not include analysis of the axons. In another species (the cat) with prominent axonal bundles, the mean number of collateral branches in the superficial layers that arose from the main trunk was 7.8 (Martin et al., 2017). Thus, although the presence of bundles indicates that the axons must run in a straight radial fashion it doesn't preclude them from having multiple branches. Our demonstration of inter-bundle distances varying systematically across the cortical surface in heat maps of tangential sections (Figure 9) also indicates a lack of uniformity in cortical minicolumns. Quantitative studies of neuronal density across different cortical areas in the chimpanzee have shown that there is a density gradient along the antero-posterior axis with the lowest density of neurons in the motor cortex, intermediate levels in the somatosensory cortex and the highest density at the occipital pole of a single individual (Collins et al., 2016). This relationship still applies when cortical thickness is taken into account as there is a 4-fold change in the numbers of neurons under a column of cortex with a surface area of 1 mm^2^ when comparing piriform cortex (45,536) with V-1 (186,232) in the marmoset (Atapour et al., 2019). In the human the range of differences in the number of neurons under 1 mm^2^ of cortex is even greater with about 10,000 neurons in the anterior piriform cortex and 100,000 in V-1 (Ribeiro et al., 2013). There are also differences across species and the number of neurons under 1 mm^2^ of cortical surface varies across different species of primate by a factor of 3 for homologous areas (Herculano-Houzel et al., 2008).

### Minicolumns in V-1 are not arranged in a regular hexagonal lattice

The regular arrangement of long parallel bundles seen in orthogonal sections of V-1 in the primate brain implies a regular geometrical arrangement of the minicolumns that they represent. By reconstructing the position of the bundles in tangential sections through layers IV and V we were able to directly study their arrangement. If all the bundles were roughly equidistant from their close neighbors, then ideally, they would be arranged in a hexagonal lattice as has been proposed in a developmental model of the orientation map (Grabska-Barwinska and von der Malsburg, 2008). When the centers of iso-orientation domains were mapped in four different species (monkey, cat, tree shrew and ferret) there was evidence for a hexagonal array (Paik and Ringach, 2011). This array may correspond to the patchy arrangement of cortico-cortical connections that are a characteristic of V-1 (Muir et al., 2011). However, this array corresponds to the centers of hypercolumns and the patch centers are about 800 μm apart. The minicolumns of this study are only about 40 μm apart and although they could also be arranged in a hexagonal lattice, as has been suggested in the somatosensory cortex (Favorov and Kelly, 1994), we were unable to find any evidence of this. When the K and G functions were plotted for the human, chimpanzee, gorilla and guinea pig reconstructions they showed that the fiber bundles were not randomly spaced but neither were they in a regular hexagonal lattice (Figure 11). Instead, when the bundle positions were connected to their closest neighbor by a short line, the connections formed twisted irregular lines that were reminiscent of the shape of orientation preference maps in V-1 (Blasdel and Salama, 1986). We conclude that part of the inter-bundle variability is caused by the irregular turning and branching of the bands where they intersect the histological section.

### Lack of uniformity in minicolumns within an individual hypercolumn

The primate V-1 usually contains prominent ocular dominance columns that form a pattern of curving branched slabs with each pair of slabs forming a hypercolumn (Hubel and Wiesel, 1977). Superimposed on these slabs are smaller cylindrical columns, which can be identified by increased levels of the mitochondrial enzyme cytochrome oxidase, which are most prominent in the supragranular layers, but are also present in layers V and VI. In the human these cytochrome oxidase blobs are oval, measure 400 by 250 μm and are organized in rows about 1 mm apart that are in register with the ocular dominance columns (Horton and Hedley-Whyte, 1984). The blobs also occur in the macaque V-1 and when the density of neurons was measured in layer III a significant difference in density was found with a higher density in the interblob regions than in the center of the blobs (Kim et al., 1997). The blobs appear darker than the interblob region when studied with myelin stains in monkeys (Horton and Hocking, 1997) but it is not clear if there is any change in density of the myelin bundles within the blobs. We were unable to stain our primate sections for cytochrome oxidase because archival material was used that had been stored in formalin for a year or more. Despite this the localized density of the fiber bundles does give an indication that there are changes in the density of minicolumns within a distance corresponding to a hypercolumn or ocular dominance column (Figure 9). This implies that adjacent hypercolumns may be composed of minicolumns that have a 10% difference in diameter. Difference of this magnitude may be associated with differences in the number or type of neuronal constituents. Analysis of the transcriptome of cortical cells in the human temporal lobe and mouse neocortex has indicated that there are at least 75 different types of neuron in the cortex with 45 of them thought to be inhibitory and 24 excitatory (with six non-neuronal) (Hodge et al., 2019). It is estimated that a typical minicolumn in macaque V-1 would contain about 30 inhibitory cells (Peters and Sethares, 1996). As the inhibitory cells migrate tangentially into the cortex during development, rather than being part of a radially arranged clone (Marin and Rubenstein, 2001) it is possible that each minicolumn has a unique combination of distinct inhibitory cells even if the complement of pyramidal cells is reasonably uniform. In practice even the complement of pyramidal cells may vary as the numbers of apical dendrites within a bundle in macaque V-1 varies between 2 and 17 and the number of axons within a bundle from 7 to 72 (Peters and Sethares, 1996). We were not able to count the number of axons per bundle in this study but the diameter of the bundles varied within a section and may indicate a similar range of axon numbers in the bundles of the great apes.

### Variation of bundle density across the extent of V-1

A surprising finding in the present study was the range of values for mean inter-bundle distances found within a single orthogonal section of V-1 in all the great apes. The smallest differences were found in the gorilla but even in this species there were significant differences between the mean inter-bundle distances for adjacent groups of 100 bundles. In the tangential sections we described density differences over a range corresponding to adjacent hypercolumns (~800 μm) but in the orthogonal sections each group of 100 bundles corresponded to a distance of about 3 mm. In one example from the chimp brain (Figure 6C) the mean inter-bundle distance rose from 28.92 to 41.27 μm (43% increase) over a distance of about 6 mm. In a human example the mean inter-bundle distance fell from 40.2 to 26.29 μm (34.6% reduction) over a distance of about 8.4 mm. There were both increases and decreases of mean inter-bundle distance over the length of section measured and so they could not have been caused by a single factor such as visual field eccentricity. Changes in the size of minicolumns across a single architectonic area have been described before (Buxhoeveden and Casanova, 2002a; Rockland and Ichinohe, 2004) but understanding the functional significance of this will require more research. More detailed information about minicolumns will be useful when relating the conservation of genetic expression and cellular morphology across species in the way that has already been shown in the motor cortex (Bakken et al., 2021).

## Conclusion

Minicolumns are represented by radial bundles of myelinated fibers that run from the base of layer III to the white matter in V-1 of the great apes (including humans) and other primates and carnivores where there may be about 20 minicolumns for each orientation column. Other mammalian orders usually do not have clear anatomical minicolumns or orientation columns in V-1 but do have other types of repeating modules or microcolumns. The total number of minicolumns or microcolumns in the primary visual cortex has a strong correlation with visual acuity and leads us to predict that measurements of visual acuity in other primates will indicate how many minicolumns are present in their V-1.

## Data availability statement

The original contributions presented in the study are included in the article/supplementary material, further inquiries can be directed to the corresponding author/s.

## Ethics statement

The studies involving human participants were reviewed and approved by National Research Ethics Service. The patients/participants provided their written informed consent to participate in this study. Ethical review and approval was not required for the animal study because only post mortem tissue was used. No living animals were involved. Written informed consent was obtained from the individual(s) for the publication of any potentially identifiable images or data included in this article.

## Author contributions

MW designed the study, made the digital reconstructions, prepared the figures, and wrote the first draft of the manuscript. OZ made the density maps, performed various nearest neighbor analyses, and helped prepare figures and text. EH and ZT sectioned and stained the brain tissue and performed data analyses while PD was responsible for the great ape post-mortems and preparation of brain blocks. LC and AP provided supervision, technical expertise and helped design the study, and acquire the brain tissue. All authors contributed to the article and approved the submitted version.

## Funding

This work was supported by the Medical Research Council [grants MC_U135097126 and MC_U135084112] and additional funding was provided by The Royal National Institute for Deaf People [grant RNID T8]. The funders had no role in study design, data collection and analysis, and decision to publish.

## Conflict of interest

The authors declare that the research was conducted in the absence of any commercial or financial relationships that could be construed as a potential conflict of interest.

## Publisher's note

All claims expressed in this article are solely those of the authors and do not necessarily represent those of their affiliated organizations, or those of the publisher, the editors and the reviewers. Any product that may be evaluated in this article, or claim that may be made by its manufacturer, is not guaranteed or endorsed by the publisher.
